# Evaluating Postural Control Functions With a Focus on Vestibular Sensation in Thalamic Astasia: A Case Report

**DOI:** 10.7759/cureus.88661

**Published:** 2025-07-24

**Authors:** Toshiya Nezu, Yuji Fujino, Kazu Amimoto, Atsushi Yamasaki, Tadamitsu Matsuda

**Affiliations:** 1 Department of Physical Therapy, Juntendo University, Tokyo, JPN; 2 Department of Rehabilitaion, Shuuwa General Hospital, Saitama, JPN; 3 Department of Rehabilitation Sciences, Sendai Seiyo Gakuin University, Miyagi, JPN; 4 Department of Physical Therapy, Bunkyo Gakuin University, Saitama, JPN

**Keywords:** mctsib, sensory interaction, sensory weighting, thalamic astasia, vestibular system

## Abstract

Thalamic astasia (TA) is a condition characterized by difficulty maintaining an upright posture due to a unilateral lesion in the thalamus. Motor paralysis, sensory disturbances, and ataxia are typically absent or mild. While vestibular dysfunction has been implicated in the onset of TA, a comprehensive evaluation strategy that considers vestibular function has yet to be established. In this report, we present a case of TA following a left thalamic infarction where the patient exhibited no abnormalities in their subjective vertical visual (SVV) function, which is often used as an indicator of vestibular function, and we discuss the potential utility of the modified Clinical Test of Sensory Interaction and Balance (mCTSIB) in patients with TA.

## Introduction

Thalamic astasia (TA) is a rare neurological condition first described by Masdeu et al. [1], characterized by difficulty maintaining upright posture despite minimal or absent motor paralysis, sensory impairment, and ataxia [1, 2].

Most pathoanatomical studies localize the lesion to the lateral or posterolateral thalamus, particularly the ventrolateral (VL) and ventroposterolateral (VPL) nuclei, which relay vestibular, cerebellar, and cortical information essential for midline postural control [1, 3, 4].

However, single-case reports and small series have also demonstrated that strategic infarction or hemorrhage of medial thalamic structures, such as the mediodorsal (MD) nucleus and the intralaminar centromedian (CM) nucleus, can produce an indistinguishable clinical picture [5-7]. Collectively, these observations indicate that both lateral and medial thalamic hubs participate in an integrated vestibulo-thalamo-cortical (VTC) and cerebello-thalamo-cortical (CTC) network whose anatomical integrity is indispensable for orthostatic control.

High-resolution MRI and CT have shown that even millimeter-sized lesions within the VL/VPL, MD, or CM nuclei can disconnect VTC and CTC pathways, leading to gravitational misperception and retropulsive or contraversive falls [2, 3, 8]. Prompt, hypothesis-driven interpretation of such subtle imaging findings therefore refines lesion localization, informs the choice of vestibulo-postural rehabilitation programs, and enhances prognostic accuracy.

Vestibular function in TA has traditionally been assessed with the subjective visual vertical (SVV) [6]. Because SVV primarily interrogates utricular afferents, more circumscribed deficits in other otolith-thalamo-cortical channels may remain undetected [8]. A broader sensorimotor assessment is thus warranted.

Here, we describe the case of a patient who developed severe postural instability and gait disturbance after a left posterolateral thalamic infarction. Although the SVV was normal, vestibular impairment was clearly demonstrated with the modified Clinical Test of Sensory Interaction on Balance (mCTSIB) [9]. The preservation of SVV implies that static otolith-tilt perception remained intact; nonetheless, the pronounced instability under dynamic sensory-conflict conditions points to a breakdown in otolith-mediated linear-acceleration processing and its vestibulo-spinal integration, rather than a deficit confined to the ocular-vestibular reflex. We propose that routine incorporation of mCTSIB into the clinical evaluation of TA can unmask covert vestibular abnormalities, thereby facilitating earlier and more targeted interventions.

This case report was previously presented as a poster at the 2024 International Society of Physical and Rehabilitation Medicine (ISPRM) World Congress, held in Sydney, Australia, from June 1 to June 6, 2024.

## Case presentation

**Figure 1:**
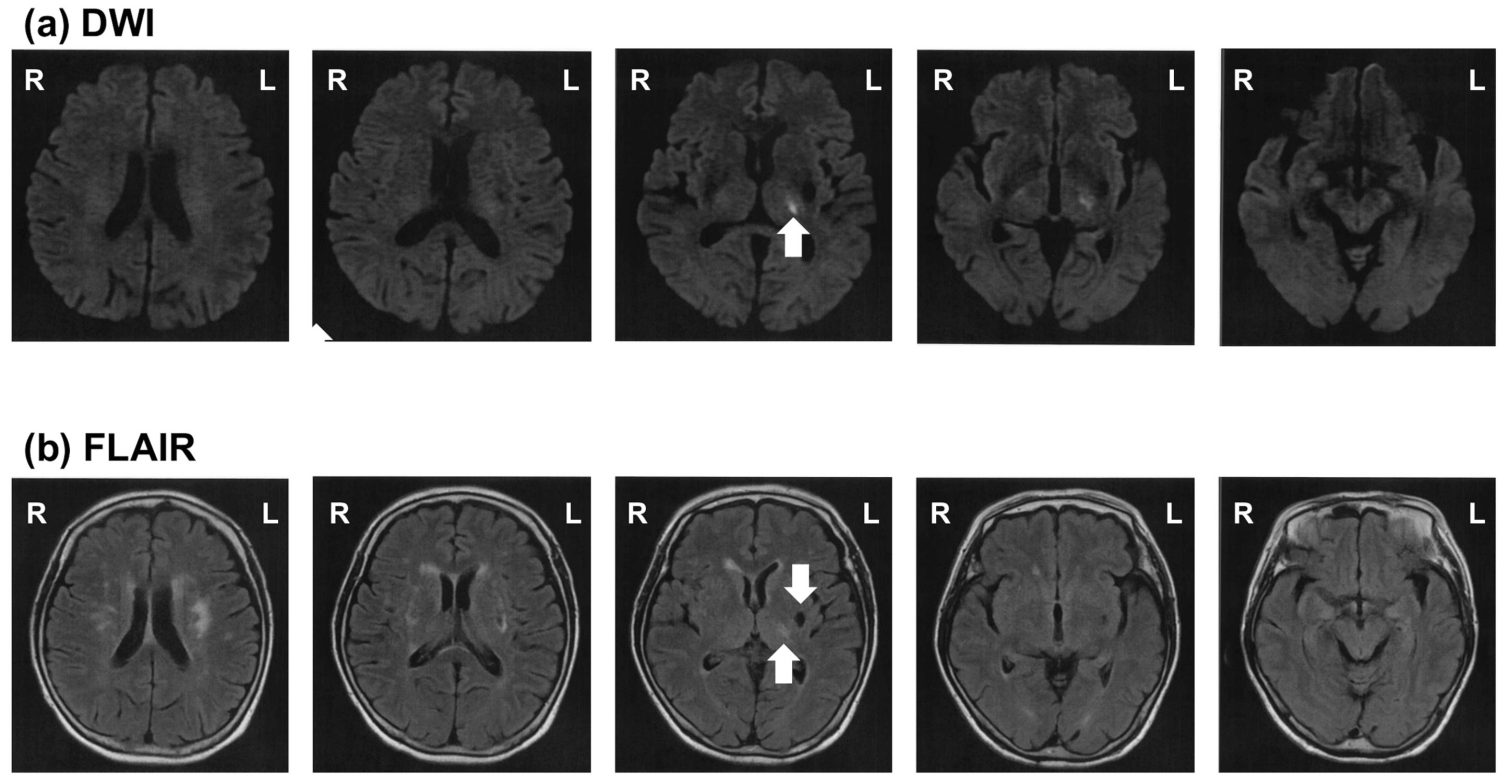
Day 1 brain MRI findings (a) Diffusion-weighted imaging (DWI) and (b) fluid-attenuated inversion recovery (FLAIR) show a high-signal lesion in the lateral part of the left thalamus (arrows). An old lacunar infarction is visible in the left basal ganglia. Axial FLAIR images were reconstructed with a slice thickness of 6.0 mm, a slice range of 7.5 mm, and an in-plane pixel size of 0.43 × 0.43 mm; the DWI sequence used the same slice thickness (6.0 mm) and slice range (7.5 mm) with an in-plane pixel size of 0.86 × 0.86 mm.

A woman in her late 60s developed acute difficulty in maintaining an upright posture and walking. Her medical history included diabetes, hypertension, and a previous left basal ganglia infarction without sequelae. Prior to the onset of symptoms, she was independent in daily life and regularly played tennis. Following the sudden onset and no observed symptom improvement, she was admitted to our hospital, where brain magnetic resonance imaging revealed an acute infarction in the left thalamus (Figures 1a, 1b). Written informed consent was obtained from the patient to publish clinical details and images in this report.

Initial evaluation

On admission (defined as Day 1), her Glasgow Coma Scale score was E4V5M6 (15/15), and she exhibited no neurological deficits affecting eye movements, speech, or swallowing. Neuropsychological assessment, including a Revised Hasegawa Dementia Scale score of 29/30 [10], confirmed that her cognitive functions were intact and that her communication abilities were preserved.

Conservative treatment was initiated, and physical therapy began on Day 2. Basic movement ability was relatively stable in the sitting position and could be performed under supervision; however, bed mobility required minimal assistance using the bed rail, and standing up and transferring required moderate assistance. In the standing position, a slight tendency to lean towards the paralyzed side was observed, and minimal assistance was required to maintain standing balance.

Subjective visual vertical for assessing vestibular function

As mentioned above, the initial neurological evaluation revealed a decline in standing ability and balance. It has been reported that a deviation of the SVV toward the opposite side of the lesion occurs in patients with TA, a symptom indicating vestibular imbalance [6]. Such abnormal tilt in the SVV has been reported to arise from either peripheral or central vestibular lesions along the vestibular pathway spanning from the labyrinth to the vestibular cortex [11]. A deviation of more than 2° from the true vertical is generally considered pathological [12]. Consequently, we decided to evaluate the patient’s SVV on the third day of hospitalization.

**Figure 2:**
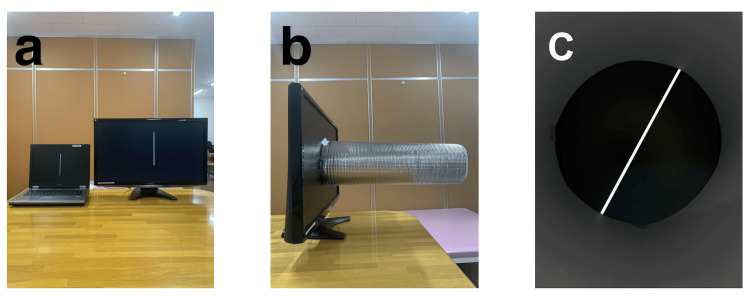
Subjective visual vertical measurement method (a) Linked by a universal serial bus (USB) cable; (b) Cylindrical tube on the personal computer (PC) screen; (c) Display on a PC screen

The measurements were carried out using computer software, a laptop, a monitor, and a 50 cm cylinder (Figure 2a). The patient was instructed to look at the computer screen through the cylinder, which was positioned to eliminate any cues for visual vertical orientation by hiding the monitor’s frame (Figure 2b). A rod was displayed on the screen, which rotated clockwise and counterclockwise at a speed of 5°/s (Figure 2c). When the patient subjectively judged the rod to be vertical, the rod rotation was stopped, and the tilt angle was measured. This procedure was performed eight times. The true vertical position was defined as 0°, and the tilt angle in either the clockwise or counterclockwise direction was defined as positive or negative, respectively.

Clinical assessment

The Stroke Impairment Assessment Scale (SIAS) was used to assess functional impairment. The SIAS is a comprehensive evaluation tool for patients with stroke that assesses motor function, sensory function, muscle tone, and joint range of motion on the affected side; motor function, language function, and visuospatial cognition on the unaffected side; as well as overall pain and trunk function [13]. Motor function on the paretic side is scored from 0 (most severe) to five (normal), whereas other items are scored from 0 (most severe) to three (normal). The total score ranges from 0 to 76, with higher scores indicating better function.

The Scale for the Assessment and Rating of Ataxia (SARA) was used to assess ataxia. The SARA encompasses eight items: gait, stance, sitting, speech, finger chase, nose-finger test, fast alternating hand movements, and heel-shin slide. Each item is rated on a scale of 0 (normal) to a maximum of four to eight (most severe, varies by item). The total score ranges from 0 to 40, with higher scores indicating more severe ataxia.

Balance was assessed using the Functional Balance Scale (FBS) and Postural Assessment Scale for Stroke (PASS). The FBS consists of 14 items, including balance in sitting and standing positions, standing from a sitting position, transfer, turning, and stepping over a step. Each item is scored on a scale of 0 (most severe) to four (normal) [14], with a total score ranging between 0 and 56. Higher FBS scores indicate better balance. The PASS encompasses a total of 12 items: five items for assessing balance in the supine, sitting, and standing positions and seven items for assessing posture conversion ability. Each item is evaluated on a four-point scale from 0 (most severe) to three (normal) [15], with a total score ranging between 0 and 36. Higher PASS scores indicate better postural control.

Trunk function was assessed using the Trunk Control Test (TCT) and Trunk Impairment Scale (TIS). The TCT is performed on a bed and is divided into four tasks: rolling from the supine position to the weak side, rolling to the strong side, sitting up from lying down, and maintaining balance in a sitting position for 30 seconds on the edge of the bed with the sole of the foot off the floor [16]. Each task is given a score of 0 points (unable to perform the task without assistance), 12 points (able to perform the task but not normally), and 25 points (able to perform the task normally). The total score is expressed as the sum of the scores of the four tasks, ranging between 0 and 100, with a higher score indicating better trunk function. The TIS [17] encompasses three subscales for static sitting balance, dynamic sitting balance, and trunk coordination. Static sitting balance is evaluated across three items with a maximum score of seven points, dynamic sitting balance across ten items with a maximum score of 10 points, and trunk coordination across four items with a maximum score of six points, resulting in a total of 17 items. The total score ranges from 0 to 23 points, with a higher score indicating better trunk function.

The Scale for Contraversive Pushing (SCP) was used to evaluate Pusher Behavior (PB) [18, 19]. The SCP evaluates three items in both sitting and standing positions: verticality in a spontaneous body posture, abduction and extension of the nonparetic extremities (pusher phenomenon), and the presence or absence of resistance to passive correction of tilted posture. Each item is given a score from 0 (no lateropulsion) to two (severe lateropulsion), with a total score ranging from 0 to six points. If any sub-item is scored > 0 or the total ≥ 1.5, it is judged to be positive for PB [20].

The walking assessment included the comfortable walking speed (CWS) and number of steps in a 10-meter walk as well as the fear of falling while walking, measured using a Visual Analog Scale (VAS) ranging from 0 (no fear of falling) to 10 (constant fear of falling).

The above evaluations were conducted on Days 3, 10, and 14, except for the 10-meter walk, which was conducted only on Days 10 and 14 due to practical limitations on Day 3.

Results

Neurological Findings on Day 3

On Day 3, the Barré sign in the upper limbs was negative, and the Mingazzini test revealed only a mild foot drop on the right side. Superficial sensation and proprioception were preserved in both upper and lower limbs. No isolated motor or sensory deficits were observed in either limb; however, the Romberg test was positive. Specifically, when asked to stand with feet together and arms at the sides, the patient maintained balance for < 5 seconds after eye closure before swaying posterior-rightward and taking a compensatory step, meeting our criterion for a positive response (< 30 s without stepping). In addition, the average SVV was 0.70° with a standard deviation of 1.54, indicating no significant deviation [12].

Clinical Assessment Results

The results of each evaluation are listed respectively in the order of Days 3, 10, and 14. The SIAS score improved from 63 to 69 and then remained stable at 69, indicating slight improvement, particularly in muscle tone. The SARA score improved from 12 to 10.5 to 7.5, indicating improvement in items related to standing and walking. Regarding balance assessment, the FBS scores were 20, 31, and 43, and the PASS scores were 26, 28, and 31 (Table 1). Regarding trunk function, the TCT scores were 87, 100, and 100, and the TIS scores were 10, 18, and 18, showing improvement from Day 3 to Day 10, but no improvement from Day 10 to Day 14.

**Table 1:** Progress from Day 3 to Day 14 SIAS: Stroke Impairment Assessment Set; FBS: functional balance scale; mCTSIB: modified Clinical Test for Sensory Interaction on Balance; EO firm: eyes opened while standing on the floor; EC firm: eyes closed while standing on the floor; EO foam: eyes opened while standing on a cushion; EO foam: eyes closed while standing on a cushion; CWS: comfortable walking speed; SARA: Scale for the Assessment and Rating of Ataxia; TCT: Trunk Control Test; TIS: Trunk Impairment Scale; VAS: Visual Analog Scale

Domain	Measure	Day 3	Day 10	Day 14	
Motor paralysis	Barré sign	Negative	Negative	Negative
Motor paralysis	Mingazzini sign	Positive	Positive	Positive
Motor paralysis	SIAS motor: upper proximal	4	4	4
Motor paralysis	SIAS motor: upper distal	4	4	4
Motor paralysis	SIAS motor: lower proximal (hip)	4	4	4
Motor paralysis	SIAS motor: lower proximal (knee)	4	4	4
Motor paralysis	SIAS motor: lower distal	4	4	4
Motor paralysis	SIAS (total)	64	69	69
Ataxia	SARA (items 5–8)	3	2.5	2.5
Sensory	SIAS light touch: R lower limb	3	3	3
Sensory	SIAS position sense: R lower limb	3	3	3
Trunk control	TCT	87	100	100
Trunk control	TIS	10	18	18
Trunk control	SCP sitting (posture/push/resist)	0.25/0/0	0/0/0	0/0/0
Trunk control	SCP standing (rosture/push/resist)	1/0/0	0/0/0	0/0/0
Vestibular function	SVV (average ± SD)	1.67±1.54	NA	NA
Vestibular function	Dizziness	Negative	Negative	Negative
Vestibular function	Nystagmus	Negative	Negative	Negative
Vestibular function	mCTSIB: EO firm	NA	30	30
Vestibular function	mCTSIB: EC firm	NA	30	30
Vestibular function	mCTSIB: EO foam	NA	30	30
Vestibular function	mCTSIB: EC foam	NA	0	8
Balance	Romberg test	Positive	Negative	Negative
Balance	Standing balance (SARA)	3	1	1
Balance	FBS	20	31	43
Balance	PASS	26	28	31
Gait	Gait (SARA)	7	6	3
Gait	10‑m CWS: speed	NA	0.52	0.89
Gait	10‑m CWS: steps	NA	30	22
Gait	Fear of falling while walking (VAS)	NA	10	2

The SCP scores recorded below are listed in the order “spontaneous body posture,” “use of nonparetic extremities (abduction),” and “resistance to passive correction of tilted posture.” Day 3 scores were 0.25/0/0 in the sitting position and 1/0/0 in the standing position, totaling 1.25 points. However, from Day 10 onwards, both the sitting and standing positions scored 0 points. Regarding walking assessment, the 10-meter CWS was 0.52 m/s (30 steps) on Day 10, and the VAS score for fear of falling while walking was 10. On Day 14, the 10-meter CWS improved to 0.89 m/s (22 steps), and the fear of falling while walking improved to two on the VAS (Table 1).

Additional Assessment: mCTSIB

**Figure 3:**
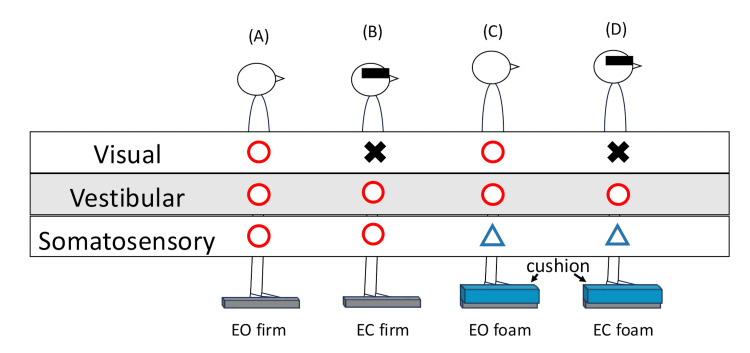
The modified Clinical Test for Sensory Interaction on Balance Our patient was tested in a shoulder-width, feet-apart stance throughout all four mCTSIB conditions. A: Standing with eyes open on the firm (EO firm); B: Standing with eyes closed on the floor (EC firm); C: Standing with eyes open on the cushion (EO foam); D: Standing with eyes closed on the cushion (EC foam). This figure is the authors’ original work.

The patient had no abnormalities in the SVV on Day 3, and there were no apparent neurological findings. However, the Romberg test was positive, and a decline in dynamic balance function, particularly during walking, was observed. Therefore, the mCTSIB was performed on Day 10 to evaluate the relative contribution of sensory modalities to postural control. The mCTSIB evaluates the ability to maintain standing posture for 30 seconds in each of the following conditions: eyes opened while standing on the floor (EO firm), eyes closed while standing on the floor (EC firm), eyes opened while standing on a cushion (EO foam), and eyes closed while standing on a cushion (EC foam); the ability to maintain a standing position for 30 s is evaluated in each case. This test identifies the primary sensory modality underlying the postural control deficit by altering the reliance on visual, vestibular, and somatosensory cues (Figure 3) [9].

Our patient, who was tested in a shoulder-width, feet-apart stance, an option reported to yield mCTSIB results comparable to the traditional feet-together position [21], was able to maintain a stable standing posture for 30 s under EO firm, EC firm, and EO foam conditions; however, she exhibited significant unsteadiness and difficulty maintaining standing balance under the EC foam condition. EC foam primarily relies on vestibular information with limited visual and somatosensory input; this suggests that the patient's impaired balance was related to vestibular dysfunction. On Day 14, the mCTSIB again revealed severe unsteadiness only under the EC foam condition, and the patient was only able to maintain a standing position for eight seconds.

Physical Therapy and Outcome

From Days 3 to 10, we focused on exercises to promote trunk and right lower limb movement, along with walking practice using parallel bars and a walker. On Day 10, the mCTSIB indicated vestibular hypofunction. A single session of vestibular-rehabilitation drills (gaze-stabilization and head-movement exercises) was therefore attempted but produced no immediate clinical change. Barefoot walking practice and plantar hardness-discrimination tasks, in contrast, elicited prompt improvements in balance, walking speed, and fear of falling while walking. Accordingly, from Days 10 to 14, rehabilitation was focused exclusively on interventions designed to augment plantar somatosensory input. Day 10, the patient rated her fear of falling while walking as 10 cm on a 10-cm VAS and reported “strong anxiety and the need to hold on to the therapist.” After four days of somatosensory-focused training, the VAS score had fallen to 2 cm on Day 14. Qualitatively, she stated that she felt “much less afraid,” initiated gait without hesitation, and required only standby supervision, indicating a marked reduction in fear of falling while walking. The patient’s 10-m CWS increased from 0.52 m/s to 0.89 m/s, a gain of 0.37 m/s that surpasses both the 95% minimal detectable change (MDC) of 0.13 m/s [22] and the widely accepted minimal clinically important difference (MCID) of 0.16 m/s [23], indicating a clinically meaningful improvement. Static balance also improved; every FBS item except tandem stance and single-leg stance could be performed independently or with standby supervision, whereas these two higher-level tasks still required manual assistance. Up to Day 10, the patient was transported exclusively by wheelchair; following the somatosensory intervention, she advanced to free ambulation on the ward with a standard walker from Day 14 onward and, during therapy sessions, was able to walk independently under standby supervision without any walking aid.

On Day 15, the patient was transferred to a convalescent hospital with the aim of regaining independence in activities of daily living, after which she was able to walk 1.5 km outdoors unaided. She was discharged from the hospital on Day 45 with all activities of daily living classified as “independent.”

## Discussion

In this report, we present a patient with TA who developed severe postural instability and gait disturbances following a left thalamic infarction. Despite only mild motor paralysis, sensory disturbances, and ataxia, significant vestibular dysfunction was revealed through mCTSIB, despite normal SVV findings. This case highlights the potential limitation of SVV as a standalone assessment for vestibular dysfunction in TA.

TA typically manifests as difficulty maintaining upright posture due to unilateral thalamic lesions, often with minimal associated motor or sensory deficits [1, 2]. Previous studies have emphasized vestibular dysfunction as a key etiological factor [1, 2, 4]. However, SVV, commonly utilized to evaluate vestibular function, may not detect subtle abnormalities in all TA patients [6, 7]. Our patient's normal SVV results contrasted significantly with the clear deficits identified through mCTSIB. Specifically, the patient exhibited marked instability exclusively under conditions emphasizing vestibular input (EC foam), indicating underlying vestibular impairment. 

In the mCTSIB, the patient could not maintain stance during the EO foam condition. This pattern is generally interpreted as difficulty using or integrating vestibular input when visual and accurate somatosensory information are unavailable [9]. Given the lesion in the ventrolateral thalamus, an area receiving vestibular projections and participating in multisensory integration [24], the finding likely reflects a central deficit in vestibulo-somatosensory integration rather than peripheral vestibular pathology.

The patient’s normal SVV indicates preserved perception of static head-tilt (graviception), whereas the pronounced instability under EC-foam suggests an impairment in processing otolith-mediated dynamic linear acceleration and its vestibulo-spinal integration. According to Angelaki & Cullen’s functional classification of otolith signals (static vs. dynamic components) [25], our findings therefore point to a selective deficit of the dynamic (acceleration) pathway while the static (tilt) pathway remains intact. We propose that the posterolateral thalamic lesion selectively disrupted central processing of acceleration-related otolith signals and their vestibulo-somatosensory integration, producing postural imbalance despite an unimpaired sense of static verticality.

While TA typically resolves within approximately two weeks [2], early assessment and targeted intervention may facilitate faster recovery and improved outcomes, and the importance of early and accurate identification of vestibular dysfunction in TA cannot be overstated. Vestibular dysfunction significantly impacts balance and gait, delaying functional recovery if unaddressed [2, 5]. Our findings underscore the utility of incorporating mCTSIB into clinical assessments for TA, providing clinicians with a sensitive tool to detect subtle vestibular abnormalities early in disease progression. Early identification allows targeted rehabilitation strategies emphasizing sensory reweighting and compensation, potentially enhancing recovery outcomes.

PB, which is thought to be associated with damage to the lateral posterior thalamus, is a potential sign of postural balance disorders that should be considered in the differential diagnosis. However, in this case, the scores of SCP sub-items “use of nonparetic extremities (abduction)” and “resistance to passive correction of tilted posture” were not greater than 0 [20]. This suggests that PB was not a contributing factor to the patient's postural instability. Additionally, other neurological conditions that could cause similar symptoms, such as brainstem involvement or peripheral vestibular dysfunction, were ruled out based on clinical assessments and imaging findings. Thus, the observed balance impairment was attributed primarily to vestibular dysfunction resulting from the thalamic lesion.

Despite persistent vestibular dysfunction and no improvement in motor strength, somatosensory function, or limb ataxia, the patient’s balance and walking speed improved substantially after the targeted sensory-reweighting interventions. These gains suggest that enhanced reliance on somatosensory and visual cues can compensate effectively for vestibular deficits. Previous studies have also highlighted the thalamus's role in integrating vestibular, somatosensory, and visual sensory inputs for balance control [24, 26].

Consistent with this, Nakamura et al. reported that a patient with infratentorial body lateropulsion showed immediate postural improvement when somatosensory cues were augmented, whereas interventions that relied mainly on visual cueing were less effective [27]. In our case, the lack of an immediate response to vestibular-focused exercises and the prompt gains seen after somatosensory-based training likewise led us to prioritize interventions that enhanced somatosensory input. Thus, vestibular dysfunction in TA may significantly disrupt sensory integration processes, emphasizing the clinical value of interventions targeting sensory reweighting.

This case has several limitations. First, although the mCTSIB revealed difficulty using vestibular cues, the test cannot localize the impairment to a single sensory system; no instrumented vestibular examinations, such as caloric irrigation or video head-impulse testing, were performed. Therefore, the findings should be interpreted as evidence of impaired vestibular utilization under sensory-conflict conditions rather than definitive proof of a primary vestibular lesion. Second, detailed quantitative assessments of somatosensory and visual function were not obtained, limiting our ability to describe compensation mechanisms comprehensively. Third, vestibular-focused rehabilitation was delivered over a relatively short period, preventing firm conclusions regarding long-term efficacy. Finally, because very few published reports of TA include comprehensive data on walking ability or other functional outcomes, we could not directly compare this patient’s recovery trajectory with that of other TA cases. Future studies should combine instrumented vestibular testing with extended rehabilitation protocols and multimodal sensory assessments to clarify the trajectory of recovery in patients with thalamic astasia.

## Conclusions

Although SVV is the conventional vestibular test used in TA, it can be normal even when dynamic vestibular-cue utilization is impaired. In this case, the mCTSIB detected such a relative vestibular deficit and guided a somatosensory-focused reweighting program that yielded clinically meaningful gains in balance and walking speed, surpassing established MDC and MCID thresholds. These findings show that mCTSIB provides diagnostic value when SVV is inconclusive and can inform targeted, mechanism-based rehabilitation.

Because the thalamus is a key locus of multisensory integration, assessments and interventions that consciously address sensory weighting may be essential for optimal recovery in TA. While the present results support incorporating both mCTSIB and somatosensory reweighting into routine care, they stem from a single case without instrumented vestibular testing; therefore, generalization must be cautious. The combination of instrumented vestibular testing with extended rehabilitation protocols and multimodal sensory assessments will be essential to further clarify the trajectory of recovery and optimize treatment approaches for patients with TA.
